# Prevalence of food security in the UK measured by the Food Insecurity Experience Scale

**DOI:** 10.1093/pubmed/fdab120

**Published:** 2021-04-19

**Authors:** Ursula Pool, Mark Dooris

**Affiliations:** Healthy and Sustainable Settings Unit, University of Central Lancashire, Preston PR1 2HE, UK; Healthy and Sustainable Settings Unit, University of Central Lancashire, Preston PR1 2HE, UK

## Abstract

**Background:**

Measurement of UK food insecurity has historically been inconsistent, making it difficult to understand trends. This study contributes by reporting and analysing data from a national survey conducted in line with UN Food and Agriculture Organization (FAO) recommendations and standard methods, providing an internationally comparable pre-coronavirus disease 2019 (COVID-19) snapshot of food insecurity.

**Methods:**

Data came from a nationally representative 2019 UK sample (*N* = 2000) surveyed by Ipsos-Mori. Prevalence of food insecurity was assessed using the UN FAO Food Insecurity Experience Scale. Logistic regression was used to model food insecurity in relation to geographic and socio-demographic variables.

**Results:**

Severe food insecurity was reported by 3% of the sample, an increase of 66.7% over the last directly comparable UK analysis (Gallup World Poll data from 2016 to 2018). Indication of some degree of food insecurity was reported by 14.2% of the sample and tended to be higher amongst younger age groups, those on lower incomes, and home renters (as opposed to owners). No geographic variables were significantly associated with food insecurity prevalence.

**Conclusions:**

The finding that prevalence of severe food insecurity was already increasing before the COVID-19 pandemic, across all areas of the UK, is cause for concern. Our results provide an important benchmark for assessing the impact of COVID-19 on food insecurity.

## Background

Food security is defined as a ‘situation that exists when all people, at all times, have physical, social and economic access to sufficient, safe and nutritious food that meets their dietary needs and food preferences for an active and healthy life’.^1^ Conversely, food insecurity can be characterized as the disruption of food intake or eating patterns due to lack of money or other resources.^2^ Although food insecurity can lead to life-threatening malnutrition, it is a phenomenon that exists on a continuum of severity with all points on that continuum having potential negative consequences for health and wellbeing.^3^ These range from stress caused by worrying about accessing suitable food, through micronutrient deficiencies, obesity, underweight and hunger to death.^3^ In addition to specific food-related conditions, food insecurity has been found to be negatively associated with health in general.^4^

Globally, food insecurity is a growing public health problem and predicted to continue to increase.^5^ Although rates of severe food insecurity are higher in low-income countries, food insecurity is nonetheless a public health concern globally, with an estimated prevalence of between 8% and 20% of the population in some high-income countries.^6^

In the UK, there is a lack of frequent, regular and methodologically consistent measurement of food insecurity. Therefore, not enough is known about the prevalence of food insecurity and how it may be changing over time. Prevalence of food insecurity is associated with socio-economic inequalities both between and within nations.^7^^,^^8^ Indicators, such as a rise in demand for emergency food provision,^9^^,^^10^ suggest that food insecurity may have been on the increase in the UK even before the COVID-19 pandemic. Furthermore, early evidence indicates that COVID-19 may have exacerbated the situation in the short-term.^11^ However, in order to be able to understand both the immediate and the longer-term impacts of the pandemic on food insecurity in both national and international contexts, a reliable baseline for comparison is needed.

The Food Insecurity Experience Scale (FIES) is a method developed by the United Nations Food and Agriculture Organization (UN FAO) to provide an internationally comparable estimate of the severity of food insecurity experience and is the specified measure for the Sustainable Development Goal indicator 2.1.2—‘prevalence of moderate or severe food insecurity in the population’.^12^ It consists of eight questions about respondents’ access to adequate food, focusing on self-reported experiences associated with difficulties accessing food:

During the last 12 months, was there a time when, because of lack of money or other resources:

1. You were worried you would not have enough food to eat?
2. You were unable to eat healthy and nutritious food?
3. You ate only a few kinds of foods?
4. You had to skip a meal?
5. You ate less than you thought you should?
6. Your household ran out of food?
7. You were hungry but did not eat?
8. You went without eating for a whole day?

The number of positive responses given by an individual is used to classify them into four categories of food security experience: food secure, mildly food insecure, moderately food insecure and severely food insecure. These categories can be thought of as zones on the continuum of food security.

The categories of moderate and severe are based on the items, ‘you ate less than you thought you should’ and ‘you went without eating for a whole day’. The selection of these items as benchmarks is based on the Global FIES, which is used for calibrating the results of individual analyses to enable meaningful comparison between countries.

The FIES has been incorporated into the Gallup World Poll, an annual international survey of individuals in around 150 countries, since 2014. Data pooled from 2016 to 2018 show that 5.6% of the UK population were estimated to have experienced moderate or severe food insecurity and 1.8% experienced severe food insecurity.^13^ A different UK survey (the Food and You Survey, 2018) found that 10% of the population experienced moderate or severe food security. This was a representative survey of adults in England, Wales and Northern Ireland, based on questions from the United States Department of Agriculture’s (USDA’s) Family Resources Survey, which reports in terms of food security as opposed to food insecurity.^14^ In the same survey, 80% of households reported high food security and 10% reported marginal food security. However, since there are differences in methodology, questions and thresholds, the prevalence rates are not directly comparable with the FIES. Further measurement is therefore needed to be able to construct an accurate picture of British food insecurity prevalence and trends.

This study aimed to add to the understanding of the changing patterns of food insecurity in the UK by assessing the prevalence of food insecurity using the same methods as the FIES, thus providing a nationally and internationally directly comparable snapshot of the prevalence rates in the UK. It also aimed to describe socio-demographic and geographical risk factors for food insecurity in a representative sample of the UK population.

## Methods

IPSOS-Mori was commissioned by The University of Central Lancashire to interview a nationally representative sample of 2000 mainland UK adults face-to-face as part of their weekly CAPIBUS survey (face-to-face Computer Assisted Personal Interviewing) in February 2019. The eight FIES questions were asked as part of the survey, which also collected demographic data that could be analysed as independent variables potentially related to prevalence of food insecurity. Demographic data chosen for analysis in this study based on previous associations with food insecurity included age, lifestage, education, ethnic origin, area, social grade, home ownership, income, geographical region and rurality. We added an additional geographic variable, distance from the coast, to examine whether living closer to the coast was associated with prevalence of food insecurity. Because this is not a variable that has been analysed in previous studies, to our knowledge, we included it as an initial exploration of the possibility that it might be associated. Face-to-face interviews were completed in people’s homes and the fieldwork lasted 1 week. The sampling method used in this type of survey is a form of random location sampling that ensures geographical spread and demographic representation to achieve a nationally representative sample.

## Results

Prevalence and severity of food insecurity were calculated in line with UN FAO recommendations using the Voices of the Hungry R program for weighted Rasch model estimation in RStudio.^15^ This method provides an internationally comparable measure of food insecurity prevalence by calibrating the scale for the country against the FIES global standard. The outputs from the Rasch analysis, shown in Table 1 below, were entered into an Excel template, publicly available from the UN FAO, to calculate the prevalences of moderate or severe, and severe food insecurity by calibrating the scale produced from our results to the FIES Global Standard Scale. The reason for following this procedure was to produce results that were internationally comparable and would include the ‘moderate or severe’ percentage estimate of food insecurity, which is a monitoring indicator for Sustainable Development Goal 2, Target 2.1. ‘Moderate’ and ‘severe’ are based on the questions/items ATELESS (‘you ate less than you thought you should’) and WHOLEDAY (‘you went for a whole day without eating’). The decision to use these is determined by how the items cluster on the Global scale. The position of ATELESS and WHOLEDAY on a scale containing all the items marks the thresholds for moderate and severe food insecurity respectively. The percentage of individuals above each threshold gives the two numbers (i.e. ‘moderate or severe’ represents the percentage of individuals above the ATELESS threshold, and ‘severe’ represents the percentage of individuals above the WHOLEDAY threshold).

**Table 1:** Item severities, standard errors, infits and outfits from Rasch analysis

*Item*	*Item severity*	*Standard error*	*Infit*	*Outfit*
WORRIED You were worried you would not have enough food to eat	−0.04439166	0.1502727	0.8637573	0.8037231
HEALTHY You were unable to eat healthy and nutritious food	−0.54958268	0.1435772	1.1153275	1.1806344
FEWFOOD You ate only a few kinds of foods	−1.12585227	0.1403581	1.1004700	1.1219912
SKIPPED You had to skip a meal	−0.19745827	0.1478560	0.9579623	0.9269401
ATELESS You ate less than you thought you should	−0.37660019	0.1454612	0.8636048	0.8205650
RUNOUT Your household ran out of food	0.99562315	0.1769337	1.0804769	1.1186530
HUNGRY You were hungry but did not eat	0.22667406	0.1554275	0.9256008	0.8661188
WHOLEDAY You went without eating for a whole day	1.07164023	0.1797162	1.0990428	1.2844513

**Fig. 1:**
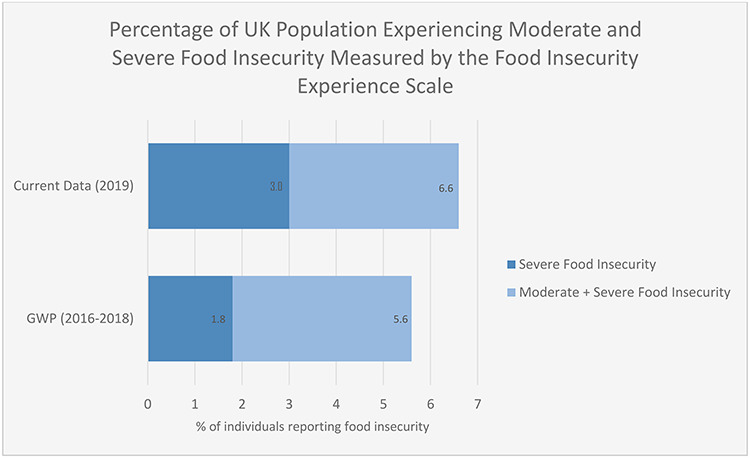
Bar chart illustrating increases in the percentages of people in nationally representative UK surveys reporting moderate+severe and severe food insecurity on the FIES comparing data from the Gallup World Poll (GWP) 2016–2018 with the data from the current study.

Mean Rasch reliability, based on an equal proportion of cases in each non-extreme raw score, was 0.69. This is acceptable, and within the range that has been reported internationally.^16^ Prevalence of any indication of experience of food insecurity (including mild food insecurity) was reported by 14.2% of individuals surveyed. Moderate or severe food insecurity was experienced by 6.6% of individuals, while severe food insecurity prevalence was reported by 3.0% of the participants. Comparing these figures with the most recent data from the Gallup World Poll (2016–2018)—which also uses the FIES, making this the most directly comparable data—suggests an increase in both moderate or severe and severe food insecurity in the UK population, as shown in Fig. 1. Although both moderate/severe and severe food insecurity have increased, the increase in severe food insecurity is proportionately greater than the overall increase, and should give cause for concern.

To explore the results further, a logistic regression model was run using IBM SPSS Statistics, Version 26, to examine the impact of 11 demographic variables on any indication of food insecurity experience in our sample (including what would be classified as mild, moderate and severe according to the Rasch analysis). Food insecurity was coded as a dichotomous variable; food secure and food insecure. Food insecurity was determined by a positive response to one or more of the eight questions comprising the FIES. We were interested in any indication of food insecurity because from a policy point of view it is necessary to understand the variables that are associated with food insecurity at all levels to be able to increase food security for all. Due to the levels of severe food insecurity in the UK (3.0% in our data) a much larger sample would have been required in order to investigate variables associated with different levels of food insecurity separately. The geographical and socio-demographic variables were: age, life stage, education, rurality, social grade, home ownership, income, gender, distance from the coast, UK region and ethnic origin. The logistic regression model was statistically significant, *ꭓ*^2^(58) = 261.5, *P* < .0001. The model explained 22.1% (Nagelkerke *R*^2^) of the variance in food insecurity and correctly classified 85.4% of cases. Sensitivity was 11.9%, specificity was 98.7%, positive predictive value was 61.4% and negative predictive value was 86.1%. Of the predictor variables, three—age, income and home ownership—were statistically significant (*P* ≤ 0.10), as shown in Table 2. Younger age groups were more likely to report food insecurity than over 60s. The 18–24 age group reported the highest levels of food insecurity, with 27.6% reporting being food insecure, as opposed to 8.2% of over 65-year-olds (odds ratio (OR) 2.45, 95% confidence interval (CI): 1.05; 5.75). Higher incomes were strongly associated with decreased prevalence of food insecurity: Compared with 29.5% of participants with an annual income below £5000 reporting food insecurity, 2.6% of participants in the £40 000–£49 999 income bracket reported food insecurity (OR 0.18, 95% CI: 0.05; 0.68).

**Table 2:** Logistic regression analysis of experience of food insecurity by socio-demographic variables

			*95% Confidence interval*	
*Variable*	**P* value*	*Odds ratio*	*Lower*	*Upper*
Age (reference 65+)	0.010			
18–24	0.039	2.453	1.047	5.747
25–34	0.010	2.801	1.284	6.111
35–44	0.000	3.517	1.797	6.885
45–54	0.003	2.350	1.348	4.095
55–59	0.019	2.113	1.134	3.938
60–64	0.168	1.572	0.826	2.993
Lifestage (reference Post-family)	0.672			
Single	0.594	1.226	0.580	2.590
Pre-family	0.773	0.881	0.371	2.093
Family	0.774	0.920	0.522	1.621
Education (reference GCSE/O-level/CSE)	0.857			
Vocational Qualifications (NVQ1 + 2)	0.699	0.902	0.533	1.525
A-level/equivalent (NVQ3)	0.333	0.805	0.519	1.249
Bachelor degree/equivalent (NVQ4)	0.093	0.652	0.396	1.075
Masters/PhD/equivalent	0.279	0.674	0.330	1.377
Other	0.475	0.796	0.425	1.490
No formal qualifications	0.426	0.817	0.497	1.343
Still studying	0.829	0.908	0.379	2.179
Do not know	0.334	0.349	0.041	2.955
Rurality (reference Metropolitan)	0.563			
Rural	0.250	1.404	0.787	2.502
Suburban	0.840	1.052	0.644	1.720
Urban	0.360	1.251	0.775	2.018
Social grade (reference A)	0.096			
B	0.473	0.679	0.236	1.953
C1	0.916	0.946	0.340	2.636
C2	0.633	0.772	0.266	2.239
D	0.873	0.916	0.311	2.697
E	0.467	1.494	0.506	4.406
Home tenure (owned/rented)	0.000	2.337	1.654	3.304
Income (reference **≤** 4499)	0.000			
4500–6499	0.021	2.864	1.169	7.016
6500–7499	0.117	2.142	0.826	5.557
7500–9499	0.080	2.105	0.914	4.845
9500–11 499	0.755	0.863	0.343	2.170
11 500– 13 499	0.309	1.578	0.655	3.799
13 500– 15 499	0.370	1.557	0.591	4.103
15 500– 17 499	0.854	1.094	0.420	2.852
17 500—24 999	0.805	0.905	0.407	2.009
25 000– 29 999	0.898	0.948	0.418	2.148
30 000– 30 999	0.448	0.731	0.325	1.645
40 000– 49 999	0.011	0.178	0.047	0.677
50 000– 74 999	0.022	0.300	0.107	0.842
75 000– 99 999	0.140	0.356	0.090	1.404
More than 100 000	0.063	0.219	0.044	1.085
Do not know	0.950	1.023	0.510	2.051
Refused	0.978	1.010	0.491	2.079
Gender (male/female)	0.743	0.954	0.719	1.266
Distance from coast (reference **≥**20 km)	0.119			
**≤**1 km	0.140	0.521	0.219	1.238
1–5 km	0.160	0.686	0.406	1.161
6–10 km	0.266	1.388	0.779	2.474
11–20 km	0.163	0.674	-.387	1.174
Region (reference Yorks & Humber)	0.799			
East Midlands	0.725	1.132	0.568	2.257
Eastern	0.446	1.291	0.670	2.486
London	0.712	1.134	0.583	2.206
North East	0.329	0.599	0.214	1.676
North West	0.762	0.895	0.435	1.839
Scotland	0.904	1.046	0.502	2.180
South East	0.695	1.149	0.574	2.299
South West	0.460	1.348	0.611	2.974
Wales	0.220	1.722	0.723	4.104
West Midlands	0.671	1.156	0.592	2.257
Ethnic origin (White/non-White)	0.780	0.942	0.621	1.430

Home ownership was associated with a lower prevalence of food insecurity: 7.9% of home owners reported food insecurity, whereas 28% of people renting their homes reported food insecurity (OR 2.34, 95% CI: 1.65; 3.30).

## Discussion

### Main findings of this study

In our representative sample of the UK adult population, 14.2% reported experiencing some degree of food insecurity in the previous 12 months. Severe food insecurity was reported by 3.0% of the sample, a relative increase of 66.7% over the previous comparable data from the Gallup World Poll (pooled data from 2016 to 2018). Food insecurity was significantly associated with income (prevalence of food insecurity tended to be higher amongst people on lower incomes), age (people in older age groups were less likely to report experiencing food insecurity) and home ownership status (people renting their homes were more likely to have experienced food insecurity than people who owned their homes). No significant association was found between geographical location and food insecurity.

### What is already known on this topic

The prevalence of food insecurity at all levels (mild, moderate or severe and severe) was found to be greater than in the most recent directly comparable analysis from the Gallup World Poll 2016–18 data.^13^ The Food and You 2018 Survey^14^ found a higher prevalence, but due to methodological differences it is difficult to make a direct comparison with these data. This highlights the importance of methodological consistency in assessing the prevalence of food insecurity. In common with other research^17^^,^^18^ socioeconomic variables, in particular income, were found to be strongly associated with food insecurity in this study. However, as previous investigations have shown, low income alone does not explain the prevalence of food security.^19^

Although there have been indications in past work that the urban/rural distinction may be associated with food insecurity,^19^ this study did not find evidence of respondents’ urban/rural location being a predictor of food insecurity. We are not aware of previous work that has investigated whether distance from the coast might be a predictive factor: however, we were interested in going beyond the urban/rural distinction and exploring the possibility that geographic variables other than those standardly used in such analyses might be relevant in the distribution of food insecurity. The coastal/inland continuum was chosen due to increasing research interest in coastal communities in the UK, which are more likely to experience higher levels of deprivation than non-coastal communities.^20^ Our analysis did not find an association between prevalence of food insecurity and proximity to the coast.

### What this study adds

The finding that severe food insecurity increased by around two-thirds is concerning. Although moderate or severe food security also increased, the majority of this increase was attributable to the increase in severe food insecurity. This suggests that severe food insecurity may be increasing more rapidly than less severe levels. It adds weight to the case for urgent policy action to address food insecurity, particularly severe food insecurity, and emphasizes the importance of more regular and consistent measurement. The results also reinforce existing knowledge about food insecurity reflecting societal inequalities.^21^ In addition, this survey data provides a nationally and internationally comparable snapshot of the prevalence of food insecurity in the UK that is more up to date than hitherto published results. It is comparable with the Gallup World Poll data for the UK and other countries, and directly compatible with the UN Sustainable Development Goals. This is the first study, to our knowledge, to include distance from the coast as a geographic variable potentially associated with food insecurity. Although we found it was not a significant predictor in this study, the geographic variables we assessed provide a baseline for tracking patterns in the prevalence of food insecurity, particularly if it changes in a geographically inconsistent manner in the future.

Particularly in the light of the COVID-19 pandemic, which has illustrated the fragility of current food systems, these results contribute to providing a baseline for comparing the experience of pre- and post-pandemic food security in the UK in future research. It is possible that the disruption to the supply chain in the early stages of the pandemic may have led to a temporary shift in the nature of food insecurity experience, from it being a phenomenon disproportionately affecting the socioeconomically disadvantaged, to a more widely dispersed experience caused by shortages of food in shops. However, preliminary research suggests this may not have affected all groups equally.^22^ It might also be expected that an economic downturn in the UK due to COVID-19 would, in the manner of other economic shocks,^12^ exacerbate existing inequalities and lead to an increase in the prevalence of food insecurity over the longer term. This highlights the importance of strengthening the resilience of food systems, particularly for vulnerable groups, and of continuing to spotlight and tackle socio-economic injustices.^23^

Although this study was planned before COVID-19, the results have taken on additional importance since the outbreak of the pandemic, which has had, and is still having, an impact on food security. Our results provide a unique point of comparison, enabling future research assessing the impact of COVID-19 on food insecurity in different geographical regions of the UK, particularly in relation to coastal communities. Without the results we report here, it would not be possible to compare the situation pre- and post-pandemic and assess whether certain categories of place have experienced differences in the prevalence of food insecurity.

### Limitations of this study

The sample in this study (*N* = 2000) was not large enough to be able to investigate some variables in depth, for example more detailed breakdowns of age, ethnic origin and geographic location. Larger sample sizes or separate dedicated studies would be required to do so. It was possible to investigate variables associated with some degree of food insecurity. However, because severe food insecurity was only experienced by 3% of the sample, the numbers were not high enough to be able to assess any potential differences in variables associated with severe food insecurity. It should not be assumed that the predictor variables will be identical for all levels of food insecurity.

Since the questionnaire relied on self-reported food insecurity, questions may be raised about the reliability of the measure. However, it is a self-report, experience-based scale by design, with reliability and validity accounted for by use of the Rasch model, and the reliability rating for the results of this study was consistent with the global scale. This is an advantage of using a globally calibrated scale such as the FIES, as opposed to other food insecurity questionnaires.

The most important findings from this study are that even before the emergence of COVID-19, food insecurity was increasing in the UK—and severe food insecurity was increasing disproportionately quickly. The results provide a pre-COVID-19 benchmark for any subsequent changes in prevalence of food security in the UK.

## Notes

**Ursula Pool**, Research Fellow **Mark Dooris**, Professor
